# Increased risk of type 2 diabetes in patients with systemic lupus erythematosus: A nationwide cohort study in Taiwan

**DOI:** 10.1097/MD.0000000000032520

**Published:** 2022-12-23

**Authors:** Yeong-Jang Lin, Chih-Chiang Chien, Chung-Han Ho, Hung-An Chen, Chao-Yu Chen

**Affiliations:** a Department of Allergy, Immunology and Rheumatology, Chi-Mei Medical Center, Yung Kang District, Tainan City, Taiwan; b Department of Nephrology, Chi-Mei Medical Center, Yung Kang District, Tainan City, Taiwan; c Department of Medical Research, Chi-Mei Medical Center, Yung Kang District, Tainan City, Taiwan.

**Keywords:** diabetes, hazard ratio, national health insurance research database, systemic lupus erythematosus

## Abstract

Data on the risk of developing diabetes in patients with systemic lupus erythematosus (SLE) are limited and have yielded mixed results. We conducted a nationwide cohort study to investigate the risk of subsequent type 2 diabetes in patients with SLE compared with matched non-SLE controls.

Data were collected from the Taiwan National Health Insurance Research Database. Adult patients newly diagnosed with SLE between 2003 to 2010 were identified as the study cohort. The non-SLE group was matched for age, gender, and date of initial diagnosis as the comparison cohort.

A total of 6159 SLE patients (87.90% female, mean age 38.79 years) were identified during this period. Of these, 206 (3.34%) developed type 2 diabetes. The 3-year incidence of type 2 diabetes was significantly higher in the SLE cohort than in the control group (130.26 vs 101.18 cases per 10,000 person-years), with an adjusted hazard ratio of 1.22 (95% confidence interval [CI] 1.04–1.44), after adjusting for age, gender, underlying comorbidities, and monthly income. Stratified analyses showed that women with SLE and low-income SLE patients (monthly income < 20,000 New Taiwan Dollar) had a higher risk of type 2 diabetes than non-SLE controls, with adjusted hazard ratios of 1.21 (95% CI 1.01–1.45) and 1.36 (95% CI 1.10–1.69), respectively.

Patients with newly diagnosed SLE had a 22% increased risk of developing type 2 diabetes during the 3-year follow-up period compared with matched controls.

## 1. Introduction

Systemic lupus erythematosus (SLE) is a systemic autoimmune disease that primarily affects women of childbearing age, and is characterized by autoantibody production, immune complex deposition, and a wide range of clinical manifestations. Patients with SLE are at an increased risk of cardiovascular disease, which is a major cause of mortality. This higher risk is multifactorial, including traditional cardiovascular risk factors, SLE disease activity, SLE-related immunological factors, and SLE-related medications.^[1]^ Diabetes is one of the modifiable cardiovascular risk factors for cardiovascular disease. Previous studies have shown an increased prevalence of diabetes in patients with SLE, ranging from 5% to 15.11%, compared with age- and gender-matched controls.^[2–4]^

However, there is a paucity of data on the risk of diabetes in newly diagnosed SLE patients, with mixed results.^[5,6]^ Hemminki et al used data from 3 Swedish health databases to estimate the risk of subsequent type 2 diabetes in patients with autoimmune diseases.^[5]^ The study included a total of 12,206 patients with SLE and found that patients with SLE had a higher risk of type 2 diabetes (standardized incidence ratio 1.83, 95% confidence interval [CI] 1.59–2.10).^[5]^ Both men and women had an increased risk of type 2 diabetes, without significant gender differences (standardized incidence ratio 1.50, 95% CI 1.09–2.02 for men and 1.95, 95% CI 1.66–2.28 for women).^[5]^ In contrast, in a nationwide population-based study using data from the UK Clinical Practice Research Datalink, 1605 patients with SLE had no increased risk of developing diabetes (adjusted hazard ratio [HR] 0.99, 95% CI 0.69–1.41 for uncomplicated diabetes and 1.01, 95% CI 0.72–1.41 for complicated diabetes).^[6]^

In this study, we used a nationwide database to assess the risk of incident type 2 diabetes in patients newly diagnosed with SLE compared with matched non-SLE controls to clarify the conflicting results in the literature.

## 2. Materials and Methods

### 2.1. Data source

This nationwide cohort study used data from the Taiwan national health insurance research database (NHIRD). The National Health Insurance Program is a nationwide health insurance system launched in 1995, and more than 99% of Taiwanese people are currently enrolled in the program. Data in this study were obtained from the longitudinal health insurance database 2000 (LHID2000), which is a representative subset of NHIRD containing records from a random sample of 1 million people in 2000 from the entire population of National Health Insurance beneficiaries. Another database acquired from the NHIRD was the Registry of Catastrophic Illness database, which included information on all 23 million Taiwanese people. Patients with proven catastrophic illnesses including SLE were exempt from copayments. The issue of a catastrophic illness certificate to patients with major rheumatic diseases was validated by rheumatologists commissioned by the Bureau of National Health Insurance by reviewing the medical records as well as laboratory and imaging studies. Because NHIRD contains de-identified secondary data released to the public for research purposes, this study was exempt from full review by the Institutional Review Board.

### 2.2. Study cohort and controls

**Figure 1.**
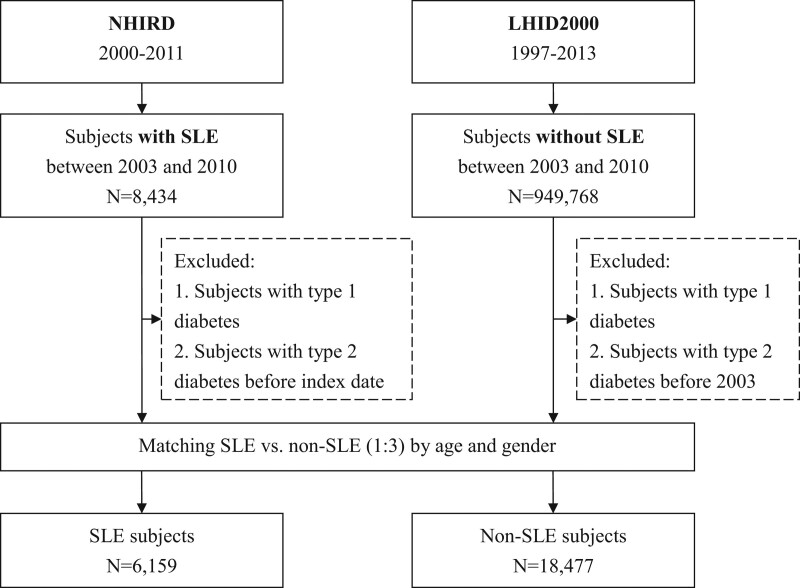
Flow diagram of the case selection process. LHID = longitudinal health insurance database, NHIRD = national health insurance research database, SLE = systemic lupus erythematosus.

Study patients were categorized into SLE and non-SLE cohorts. Patients aged ≥ 20 years newly diagnosed with SLE (international classification of diseases, 9^th^ revision, clinical modification [ICD-9-CM] code 710.0) from 2003 to 2010 were included in the study cohort. For higher diagnostic accuracy, a case of SLE was defined by the possession of a catastrophic illness certificate for SLE. The index date for the study cohort was determined as the date of the first ambulatory care visit for a diagnosis of SLE. To identify new SLE cases, those with index dates before January 1, 2003, were excluded. The comparison cohort comprised subjects without SLE (3 for every patient in the SLE cohort) randomly selected from the LHID2000 and frequency matched by age, gender, and year of the index date. To confirm new-onset type 2 diabetes, patients in both cohorts with a diagnosis of diabetes (ICD-9-CM code 250) before the index date were excluded. A flowchart of the study subject selection is presented in Figure 1.

### 2.3. Outcome assessment

The outcome of interest was new-onset type 2 diabetes (ICD-9-CM code 250.X0/X2). Patients were defined as having type 2 diabetes if the diagnosis was repeated at least 3 times either with outpatient claims or upon hospitalization. Incident type 2 diabetes was identified during the follow-up and the 3-year incidence rate was calculated. Incidence rates were estimated for each cohort separately and then stratified by age, gender, monthly income, and underlying comorbidities. The selected baseline comorbidities included hypertension (ICD-9-CM codes 401–405), hyperlipidemia (ICD-9-CM code 272), coronary artery disease (ICD-9-CM codes 410–414), cerebrovascular accident (ICD-9-CM codes 430–438), and chronic renal disease (ICD-9-CM code 585). These comorbidities were determined based on at least 1 in-patient claim or 3 out-patient claims before the SLE diagnosis. All study patients were followed up for 3 years or until December 31, 2010.

### 2.4. Statistical analysis

All statistical analyses were performed using Statistical Package for Social Sciences for Windows (version 17.0; SPSS Inc., Chicago, IL). Mean values were reported as standard deviation. Comparisons of groups with or without SLE were performed using the chi-square test for categorical variables and Student’s *t*-test for continuous variables. The adjusted HRs and 95% CIs were calculated using the Cox proportional hazard regression model to identify significant predictors of type 2 diabetes. The Kaplan–Meier method was used to estimate the cumulative incidence, and the differences between curves were tested with a 2-tailed log-rank test. All statistical tests were 2-tailed, and only associations with a *P*-value < 0.05 were considered statistically significant.

## 3. Results

Table 1 compares the demographic characteristics between the SLE and control cohorts. A total of 24,636 patients (6159 and 18,477 in the SLE and non-SLE cohorts, respectively) were sampled from the NHIRD and LHID2000, respectively. The mean age was 38.79 years, and 87.90% of the patients were female. Nearly half (47.70%) of the subjects in both cohorts were aged between 20 and 34 years. Compared with the control group, the SLE cohort had significantly lower monthly income and a higher prevalence of baseline comorbidities including hypertension, hyperlipidemia, coronary artery disease, cerebrovascular accident, and chronic renal disease (*P*-value < 0.0001).

**Table 1:** Demographic characteristics of the SLE and control cohorts.

Variable	SLE	Non-SLE	*P*-value
	n = 6159	n = 18,477	
Age (yr)	38.79 ± 14.13	38.79 ± 14.13	1.0000
Age subgroup			1.0000
20–34	2938 (47.70)	8814 (47.70)	
35–49	2015 (32.72)	6045 (32.72)	
50–64	801 (13.01)	2403 (13.01)	
≥65	405 (6.58)	1215 (6.58)	
Gender			1.0000
Female	5414 (87.90)	16242 (87.90)	
Male	745 (12.10)	2235 (12.10)	
Monthly income (NTD)			< .0001
<20,000	3669 (59.57)	10457 (56.59)	
20,000–40,000	1884 (30.59)	5862 (31.73)	
>40,000	606 (9.84)	2158 (11.68)	
Underlying comorbidity			
Hypertension	661 (10.73)	1004 (5.43)	< .0001
Hyperlipidemia	192 (3.12)	310 (1.68)	< .0001
Coronary artery disease	162 (2.63)	224 (1.21)	< .0001
Cerebrovascular accident	176 (2.86)	138 (0.75)	< .0001
Chronic renal disease	94 (1.53)	38 (0.21)	< .0001

Data are expressed as n (%) or mean ± SD.

NTD = New Taiwan Dollar, SLE = systemic lupus erythematosus.

Table 2 shows the incidence of type 2 diabetes in subjects with and without SLE. In both cohorts, the incidence by age stratum increased with older age. In addition, the incidence rate was higher in men than in women. Of the 6159 SLE patients, 206 (3.34%) developed type 2 diabetes. The 3-year incidence of type 2 diabetes was higher in the SLE cohort than in the non-SLE cohort (130.26 versus 101.18 cases per 10,000 person-years), with an adjusted HR of 1.22 (95% CI 1.04–1.44), after adjustment for age, gender, baseline comorbidities, and monthly income. Stratified analyses showed that women with SLE and low-income SLE patients (monthly income < 20,000 New Taiwan Dollar) had a higher risk of type 2 diabetes than non-SLE controls, with adjusted HRs of 1.21 (95% CI 1.01–1.45) and 1.36 (95% CI 1.10–1.69), respectively. Other stratified analyses, including age and underlying comorbidities, did not show statistically significant differences.

**Table 2:** The 3-year incidence of type 2 diabetes in subjects with and without SLE and the Cox model estimated hazard ratio for patients with SLE.

	SLE (n = 6159)			Non-SLE (n = 18,477)			Adjusted HR (95% CI)	*P*-value
	Type 2 diabetes n (%)	PYs	Rate*	Type 2 diabetes n (%)	PYs	Rate*		
Overall analysis	206 (3.34)	15814.33	130.26	507 (2.74)	50,108.93	101.18	1.22 (1.04–1.44)	.0179
Stratified analysis								
Age (yr)								
20–34	42 (1.43)	7805.24	53.81	100 (1.13)	24,268.34	41.21	1.23 (0.85–1.78)	.2672
35–49	71 (3.52)	5210.63	136.26	155 (2.56)	16,491.81	93.99	1.29 (0.97–1.72)	.0817
50–64	53 (6.62)	1966.01	269.58	144 (5.99)	6324.26	227.69	1.05 (0.76–1.45)	.7887
≥65	40 (9.88)	832.45	480.51	108 (8.89)	3024.52	357.08	1.20 (0.83–1.74)	.3374
Gender								
Female	168 (3.10)	14022.09	119.81	419 (2.58)	44,128.54	94.95	1.21 (1.01–1.45)	.0425
Male	38 (5.10)	1792.24	212.03	88 (3.94)	5980.39	147.15	1.32 (0.89–1.96)	.1623
Monthly income (NTD)								
<20,000	130 (3.54)	9448.11	137.59	281 (2.69)	28,717.17	97.85	1.36 (1.10–1.69)	.0041
20,000–40,000	57 (3.03)	4773.42	119.41	170 (2.90)	15,592.44	109.03	1.00 (0.74–1.35)	.9822
>40,000	19 (3.14)	1592.80	119.29	56 (2.59)	5799.32	96.56	1.18 (0.70–2.01)	.5339
Comorbidity								
Hypertension	59 (8.93)	1501.91	392.83	103 (10.26)	2514.65	409.60	1.09 (0.78–1.52)	.6025
Hyperlipidemia	15 (7.81)	431.87	347.33	32 (10.32)	775.62	412.57	1.03 (0.54–1.97)	.9228
Coronary artery disease	14 (8.64)	338.76	413.27	23 (10.27)	557.07	412.87	1.04 (0.52–2.09)	.9126
Cerebrovascular accident	19 (10.80)	358.18	530.47	15 (10.87)	334.11	448.95	1.79 (0.86–3.73)	.1209
Chronic renal disease	6 (6.38)	180.16	333.04	4 (10.53)	83.04	481.67	0.62 (0.14–2.87)	.5421

CI = confidence interval, HR = hazard ratio, NTD = New Taiwan Dollar, PYs = person-years, SLE = systemic lupus erythematosus.

* Per 10,000 person-years.

**Figure 2.**
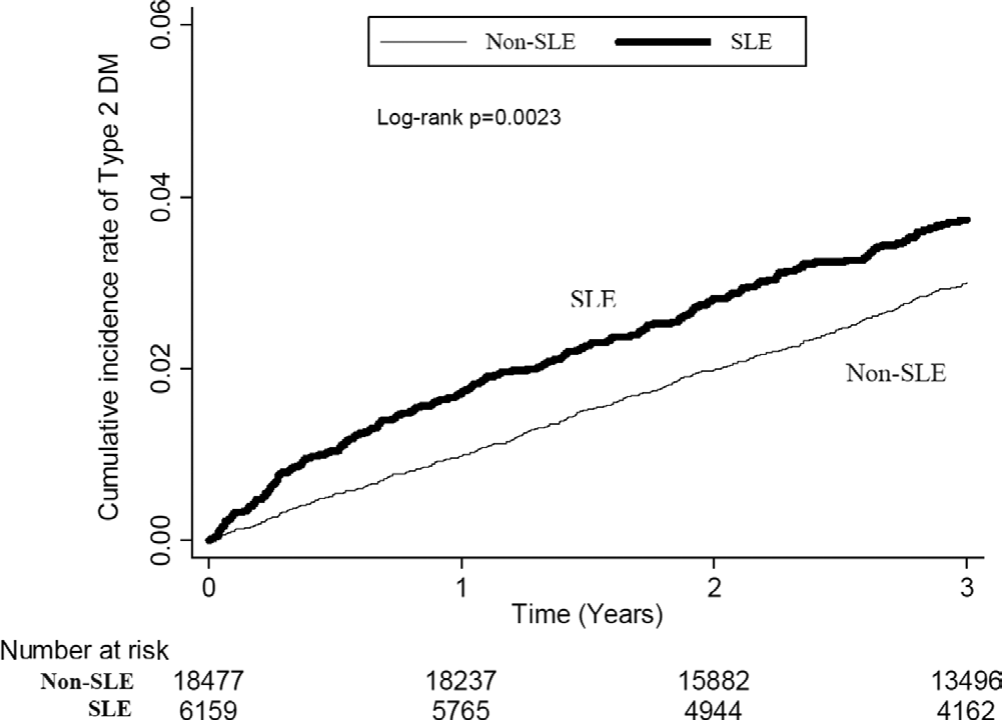
Cumulative incidence of type 2 DM in subjects with and without SLE using the Kaplan–Meier method. DM = diabetes mellitus, SLE = systemic lupus erythematosus.

Figure 2 shows the cumulative incidence of type 2 diabetes in subjects with and without SLE during a 3-year follow-up using the Kaplan–Meier method. Log-rank test analysis showed that SLE patients had a significantly higher incidence of type 2 diabetes than non-SLE patients (*P*-value = 0.0023).

## 4. Discussion

This study used a national database to examine the risk of developing type 2 diabetes in newly diagnosed SLE patients. Our study showed that patients with SLE had a 22% higher risk of type 2 diabetes within 3 years compared with matched controls.

The higher risk of type 2 diabetes in patients with SLE might be due to increased insulin resistance caused by SLE itself and medications used to treat SLE. Insulin resistance plays a major role in the development of glucose intolerance and type 2 diabetes.^[7]^ In a study of 44 nondiabetic women with SLE, the incidence of insulin resistance was higher in patients with SLE than in age-matched controls.^[8]^ Although patients using steroids had lower insulin sensitivity, insulin sensitivity was still significantly reduced in those who did not use steroids compared with controls.^[8]^ A cross-sectional study in Spain reported higher rates of insulin resistance in patients with SLE compared with controls.^[9]^ This difference remained statistically significant after adjustment for classical cardiovascular risk factors and prednisone use.^[9]^ These findings suggested that SLE independently increases insulin resistance, which over time progresses to type 2 diabetes.

Medications used to treat SLE, including glucocorticoids and calcineurin inhibitors, are associated with an increased risk of diabetes. Glucocorticoids are frequently used to treat SLE owing to their potent anti-inflammatory and immunosuppressive effects. Glucocorticoids are known to cause hyperglycemia through a variety of mechanisms, including inducing or worsening preexisting insulin resistance, increasing hepatic gluconeogenesis, causing islet cell dysfunction, and, in the long term, stimulating appetite and weight gain.^[10,11]^ A UK study reported that glucocorticoid use was associated with a strong dose-dependent increase in the incidence of type 2 diabetes in SLE patients, regardless of body mass index, family history of diabetes, or disease duration, even after adjustment for systemic inflammatory activity.^[12]^ Calcineurin inhibitors (e.g., tacrolimus, cyclosporine, and voclosporin) are commonly used in induction and maintenance therapies for lupus nephritis. Calcineurin inhibitors can cause glucose intolerance and lead to diabetes by reducing insulin secretion and increasing insulin resistance.^[13]^ Therefore, regular diabetes risk assessment and screening are essential during glucocorticoid and calcineurin inhibitor therapy, regardless of the prescribed dose.

In this study, women with SLE had a significantly higher risk of type 2 diabetes than non-SLE controls. Men with SLE also had a higher incidence of type 2 diabetes than controls. However, this difference did not reach statistical significance (adjusted HR 1.32, 95% CI 0.89–1.96). SLE typically affects women at far higher rates than men. However, males were associated with more active disease at diagnosis, regardless of age or ethnicity.^[14]^ A study found a gender difference in glucocorticoid use in patients with SLE; the frequency and dose of glucocorticoids were significantly higher in male SLE patients.^[15]^ Therefore, men with SLE are more likely than women to be treated with high-dose glucocorticoids, which may lead to the development of insulin resistance or type 2 diabetes. The gender difference in diabetes risk in our study may be due to the relatively small sample size of males with SLE, which results in a wider confidence interval with a larger margin of error. Further research is needed to understand the effect of the male gender on diabetes risk in patients with SLE.

In the present study, low-income SLE patients had a significantly higher risk of type 2 diabetes than non-SLE controls. Previous studies have shown that low income is independently associated with higher morbidity, increased damage accumulation, and worse prognosis in patients with SLE.^[16,17]^ In a longitudinal study of the Hopkins Lupus Cohort, low income was also associated with cardiovascular risk factors (including diabetes), myocardial infarction, and stroke compared with individuals in the highest income category.^[18]^ It is well known that low income is associated with a higher incidence of diabetes and diabetes-related complications.^[19]^ The mechanisms by which low income is associated with type 2 diabetes are not clear. Our findings suggest that low-income SLE patients are at a particularly high risk of developing diabetes, emphasizing the importance of regular diabetic risk assessment and testing in these patients.

Because of the increased risk of developing type 2 diabetes in patients with SLE, effective screening and monitoring strategies should be employed for prevention and timely detection. Guidelines for the management of SLE recommend screening for diabetes at baseline and at least annually thereafter.^[20]^ Fasting plasma glucose and glycated hemoglobin levels should be tested regularly to screen for prediabetes or diabetes. Studies have shown that lifestyle interventions such as a healthy diet, regular exercise, and weight control in high-risk individuals are effective in preventing type 2 diabetes and are considered first-line preventive therapies.^[21,22]^ Furthermore, it is well known that glucocorticoid use increases the risk of diabetes; therefore, in patients with SLE, it is strongly recommended to reduce steroid exposure and use steroid-sparing agents to minimize adverse effects.^[23]^ Hydroxychloroquine, an antimalarial drug, is widely used to treat SLE. Hydroxychloroquine has been shown to reduce the incidence of diabetes in patients with SLE.^[24]^ Patients with higher cumulative doses (≥ 129 g) had a significantly lower risk of diabetes than patients not receiving hydroxychloroquine.^[24]^ A Canadian cohort study also found that patients with SLE who adhered to antimalarial therapy had a 39% lower risk of developing type 2 diabetes than those who discontinued therapy.^[25]^ However, the protective effect was lost when less than 90% of the prescribed dose was taken.^[25]^

This study has 3 advantages. First, it is a national, population-based study using an administrative database that removes selection bias. Second, the presence of a large number of study subjects and age- and gender-matched controls can estimate the risk of type 2 diabetes in patients with SLE. Third, more than 98% of Taiwan’s population is Han Chinese. Homogenous populations reduce the confounding effects of ethnicity.

Our study has several limitations. First, using ICD codes from claims data to diagnose SLE, type 2 diabetes, and other comorbid medical conditions may impair accuracy. To address this concern, for the SLE cohort, we only recruited subjects with a catastrophic illness certificate for SLE, which would minimize the probability of recruiting non-SLE patients. Likewise, subjects were considered to have type 2 diabetes only if the diagnosis was repeated at least 3 times at the time of outpatient claims or hospitalization. Thus, the possibility of miscoding errors was minimized in this study. Second, information about family history of diabetes, weight and height, physical activity status, smoking habits, alcohol consumption, and education level is not recorded in NHIRD, which may also influence diabetes risk. Therefore, the variables used for adjustment may be limited.

## 5. Conclusion

Patients with newly diagnosed SLE had an increased risk of developing type 2 diabetes compared with matched controls. Therefore, diabetes screening and prevention strategies should be implemented to improve SLE care.

## Acknowledgments

The authors would like to thank the Taiwan National Health Research Institute for permission and authorization to use this dataset. This study does not represent the official viewpoint of the Taiwan National Health Research Institute.

## Author contributions

**Conceptualization:** Yeong-Jang Lin, Chih-Chiang Chien, Hung-An Chen.

**Data curation:** Chung-Han Ho.

**Investigation:** Yeong-Jang Lin, Chih-Chiang Chien, Chung-Han Ho, Chao-Yu Chen.

**Methodology:** Yeong-Jang Lin, Chih-Chiang Chien, Chung-Han Ho, Hung-An Chen.

**Software:** Chung-Han Ho.

**Writing – original draft:** Yeong-Jang Lin.

**Writing – review & editing:** Yeong-Jang Lin.
